# Depression and Cognitive Impairment in Disability-Free Early Multiple Sclerosis

**DOI:** 10.1155/2003/843760

**Published:** 2003-01-01

**Authors:** Claus G. Haase, Michael Tinnefeld, Marc Lienemann, Reinhard E. Ganz, Pedro M. Faustmann

**Affiliations:** ^1^Department of NeurologyUniversity Hospital EssenGermany; ^2^Department of Neuroanatomy and Molecular Brain ResearchRuhr-University BochumGermany; ^3^Department of NeurologySt. Johannes HospitalHagenGermany; ^4^Swiss Epilepsy CenterZurichSwitzerland; ^5^Institute of Clinical PharmacologyBayer Pharma Research CenterWuppertalGermany

**Keywords:** multiple sclerosis (MS), depression, benign course, cognition

## Abstract

Cognitive and emotional capabilities were evaluated in 73 female patients with stable relapsing-remitting definite, and/or laboratory-supported multiple sclerosis (MS) and were compared with 32 matched healthy controls. Patients were categorized according to their score in the expanded disability status scale (EDSS) to either no (EDSS 0, *n* = 33) or few clinical signs (EDSS 1–2, *n* = 40) of MS without physical disability. Patients with EDSS > 0 were characterized by significantly (p These results indicate that depression may present as an early sign in MS followed by cognitive impairment, in particular visuo-spatial short-term memory, before physical disability appears. Neuropsychological tests as mentioned here could serve as early diagnostic tools to detect subtle disease progression and to initiate and monitor disease modifying therapies.

Patients with EDSS > 0 were characterized by significantly (*p* < 0.001) higher scores on “von Zerssen’s“ depression scale, compared to controls. Patients with higher EDSS scores (1–2) showed significantly decreased performance with respect to the total score of Kimura’s Recurring-Figures-Test (*p* < 0.001), in addition. Regarding visuo-constructive functioning, patients with EDSS = 0 performed to a significantly lower level (*p* < 0.001), compared to controls.

## 1. Introduction

Demyelinating inflammatory central nervous diseases, like multiple sclerosis (MS), are associated with impairment of neuropsychological functions, including memory and mood [21,29,31]. However assessment of cognitive decline in MS is often limited due to high variability of its degree and different aspects of test-systems used as well as to the high variability of individual course, grade of impairment and relapse frequency [14]. While most studies have been performed in physically disabled patients (EDSS $> 2$, according to Kurtzke's Expanded Disability Status Scale [18]) with long-standing disease, which revealed a wide range of cognitive and affective disturbances, no psychometric studies in MS patients without disabilities (EDSS $< 2$) were carried out, so far. In the affective realm of MS e.g., depressive states were found to be more frequent than manic symptoms [10,19,20,22,24–26,29–32,38]. With regard to memory performance, already disease duration of less than 2 years was found to result in memory dysfunction including visual, verbal and short term memory [14,23,29]. In particular short-term memory dysfunction has been described to affect visuo-spatial more severely than verbal capabilities [13,21,23,26,

[1]Dedicated to Dr. Klausdieter Segerath in occasion of his 75th birthday.
[2]Both authors contributed equally to this work.
*Corresponding author: Pedro M. Faustmann, Faculty of Medicine, Department of Neuroanatomy and Molecular Brain Research, Ruhr-University of Bochum, Universitätsstr. 150, D-44780 Bochum, Germany. Tel.: +49 234 32 24405; Fax: +49 234 32 14089; E-mail: pedro.m.faustmann@ruhr-uni-bochum.de.

ISSN 0953-4180/03/$8.00 © 2003 – IOS Press. All rights reserved

31]. Kimura's Recurring Figures Test [15,17] has previously been proven to measure very sensitively visual memor deficits in MS patients [12].

The present study supplements prior published results and examines whether cognitive and affective disturbances, like depression, can already be found in early MS patients who are not yet physically disabled [9].

## 2. Methods

### 2.1. Subjects

In order to certify a homogeneous study population only women have been included into the study. Not only are women more frequently affected by MS (2–3:1) and represent a higher proportion of patients with relasping-remitting MS, but for technical reasons female patients have a stronger attitude to participate early and continuously in ambulatory health care than men. Therefore men show higher EDSS scores than female patients on average and at first contact, which was an exclusion criterion for participation in this study, a low EDSS.

Seventy-three female patients (mean age: 31 years, range: 17–40 years) who were diagnosed a relapsing remitting clinically definite or laboratory-supported definite MS [28] were included into the study. All patients had a stable long-standing MS (duration: $63 + / - 51$ months) without physical disability (EDSS 0–2, median 1, mean 1.14). In order to evaluate the dynamic relation between physical, cognitive and emotional impairment, we formed two groups of patients: MS group 1, $\text{EDSS} = 0$ ($n = 33$) and MS group 2, $\text{EDSS} = 1 - 2$ ($n = 40$), and compared them with thirty-two matched healthy women as normal controls (NC).

No subject had a history of psychiatric or neurologic disorder or was on medication at the time of testing. All subjects had normal or corrected-to-normal visual acuity, and had been free of medication affecting the central nervous system, including corticosteroids for at least 4 weeks prior to the examination, in order to minimize pharmacological influences on test results. All persons had at least secondary school education and gave informed consent to participate in this study. Demographic variables are summarized in Table 1.

For intelligence assessment all subjects completed a short version of the German Wechsler Adult Intelligence Scales [5]. This version is based on performance on the subtests: General knowledge, similarities, picture completion and block design. The block design (DS) subtest was excluded for determining the general intellectual functioning, since it puts MS patients at a disadvantage because of the motor speed demands [27,29]. IQ-scoring was computed as the mean of the IQs based on general knowledge, similarities and picture completion, revealing no differences between patients and healthy controls with regard to age and IQ ($p > 0.23$) (Table 1).

### 2.2. Affective measurements

Von Zerssen's deppression scales are self-rating scales with two parallel forms to calculate the mean value [33–36]. We preferred the Zerssen-scales over Beck's-Depression-Inventory (BDI) [2], in order to cover three different aspects of depression by scaling: depression (DS), somatic/physical complaints (BL) and the actual mood (BFS) at time of testing.

Self reported depression was assessed by the von Zerssen's parallel depression scales: D-S and D-S'. These scales evaluate psychological manifestation of depression and/or anxiety as a mental aspect of persisting depression and delivers information on the extent of a depressive, anxious or short-tempered (dysphoric) mood which goes beyond the more strictly used term of depression measured by the BDI [2]. To assess physical and general discomfort at time of examination the physical complaint scale (B-L/B-L', parallel forms) was used to quantify subjective impairments resulting from actual somatic or general complaints. The sum value, BL, illustrates the patient's somatic complaints regardless their causes. Self reported actual mood was assessed by von Zerssen's parallel scales Bf-S and Bf-S', a unidimensional self-rating mood scale.

### 2.3. Cognitive measurements

To assess the visuospatial/visuoconstructive functioning, the block design test was used, as part of the standardized Wechsler Intelligence Scale (WIP). The sum of points achieved were transformed into IQ-values, afterwards the variable MT was the extracted and analyzed.

The visuospatial short-term memory was assessed by nonverbal Kimura's Recurring-Figures Test (RFT), (Kimura [17], validated as a German version by Hartje [15]) with two sorts of abstract drawings: geometric and nonsense ("spaghetti") drawings. Every card is shown to the patient for a maximum of three seconds and should be stored in memory. Some of the drawings are shown repeatedly. These are the

Table 1
Subject group characteristics (means and SDs)

| | NC controls | MS(1) EDSS $= 0$ | MS(2) EDSS $= 1-2$ | Total |
|---|---|---|---|---|
| n | 32 | 33 | 40 | 105 |
| Age (mean, SD) | 31.03 | 30.73 | 31.35 | 31.06 |
| | (6.03) | (5.71) | (4.95) | (5.49) |
| IQ (mean, SD) | 104.40 | 101.27 | 101.86 | 102.46 |
| | (5.41) | (9.68) | (9.10) | (8.38) |

Table 2
Results for DS, BL and BFS (means and SDs)

| | NC | MS(1) | MS(2) |
|---|---|---|---|
| DS | 4.95 (4.30) | 7.49 (8.17) | 10.21 (6.66) |
| BL | 11.30 (7.16) | 18.85 (13.04) | 23.03 (13.21) |
| BFS | 9.5 (7.40) | 13.50 (13.77) | 15.10 (11,35) |

"recurrent figures". In case of remembering such a drawing, the person should react verbally. Three variables have been extracted: NETGEOM, NETNONS and NET. NETGEOM is the difference of the correctly and misleadingly remembered geometric recurrent figures, NETNONS is the difference of the correct and the misleadingly remembered nonsense recurrent figures. The performance of the visuo-spatial short-term memory, NET, was determined as Kimura's „total netscore". NET is the sum of NETGEOM and NETNONS, therefore the difference of all correct and misleadingly remembered recurrent figures.

## 3. Statistics

Statistical analyses were performed using SPSS version 10. Group differences were evaluated by analysis of variance (ANOVA, F). If there were significant group differences in variances, nonparametric tests (Kruskal-Wallis ANOVA, H) were used. Post-hoc paired group comparisons were carried out using the strict Bonferroni's alpha correction or, in case of nonparametric analyzing, Mann-Whitney U-tests, respectively. Significance was considered, if $p < 0.05$.

## 4. Results

### 4.1. Affective variables

The means and standard deviations (SD) for depression (DS), somatic/physical complaints (BL) and mood (BFS) are presented in Table 2.

26% of all MS patients had a clinical significant level of depression ($>= 90.$ percentile), i.e. 15% in MS(1) and 35% in MS(2).

Analyses of variances revealed significant differences for DS between all three groups ($F(2, 102) = 5.688, p = 0.005$). Post-hoc Bonferroni's comparisons showed that the more "disabled" patient group MS(2) scored significantly higher on depression than the control group NC ($p = 0.003$). With regard to physical complaints (BL) 26% of the whole MS sample had a clinical significant quantity of physical complaints ($>= 90.$ percentile), 18% in MS(1) and 33% in MS(2). The three groups differed significantly ($H = 15.86$, $df = 2$, $p < 0.001$). The MS group 1 and MS group 2 reported to have significantly more physical complaints in comparison to the control group ($U = -2.38$, $p = 0.017$ and $U = -3.96$, $p < 0.001$, respectively). Actual mood did not reveal differences in the BFS scores ($H = 3.76$, $df = 2$, $p = 0.15$). Performance is depicted in Fig. 1.

### 4.2. Cognitive variables

In the visuo-constructive functions (block design) test control subjects scored an average IQ of 120.69 ($SD = 12.02$); the MS(1) and MS(2) groups reached IQ-scores of 108.97 ($SD = 13.98$) and 109.0 ($SD = 16.05$), respectively (see Fig. 2). The statistical analysis of the block design IQ scores yielded a significant difference between groups ($H = 16.08$, $df = 2$, $p < 0.001$). This significant difference was due to a decreased level of visuo-constructive functions ability relative to the NC group for both the MS(1) ($U = -3.55$, $p < 0.001$) and the MS(2) group ($U = -3,46$, $p < 0.001$) (Fig. 2).

Visuospatial memory assessment tested by the number of items remembered in Kimura's RFT are presented in Table 3.

Analyses of variance revealed significant differences for the RFT scores in the recognition of nonsense figures (NETNONS: $F = 6.18$, $df = 2$, $p = 0.003$). The three groups did not differ significantly on the number of remembered (R-F) geometric recurring figures (NETGEOM: $H = 2.85$, $df = 2$, $p = 0.24$).

Analysis of the number of remembered nonsense figures yielded significant group differences (NET-

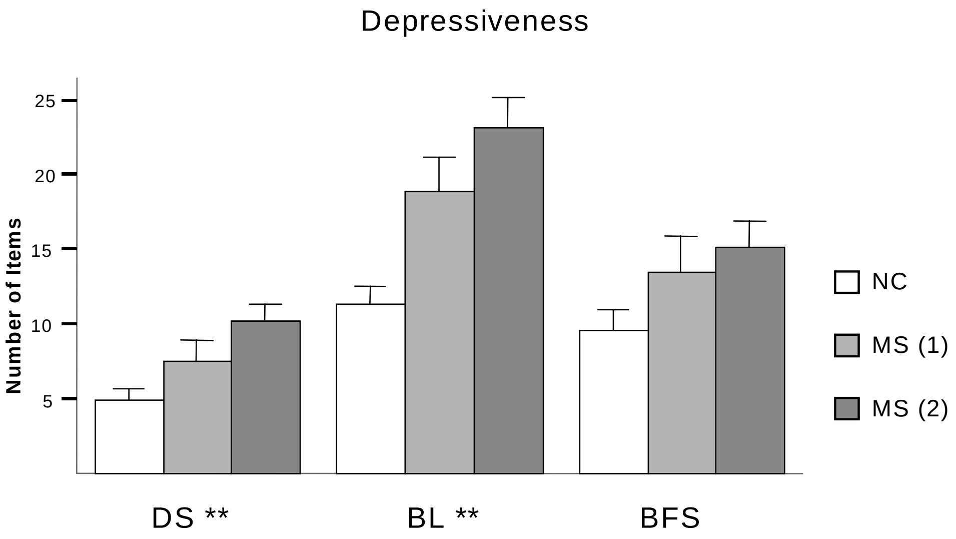

Fig. 1. Mean number (+/− SEM) of depression (DS), somatic/physical complaints (BL) and actual mood (BFS).

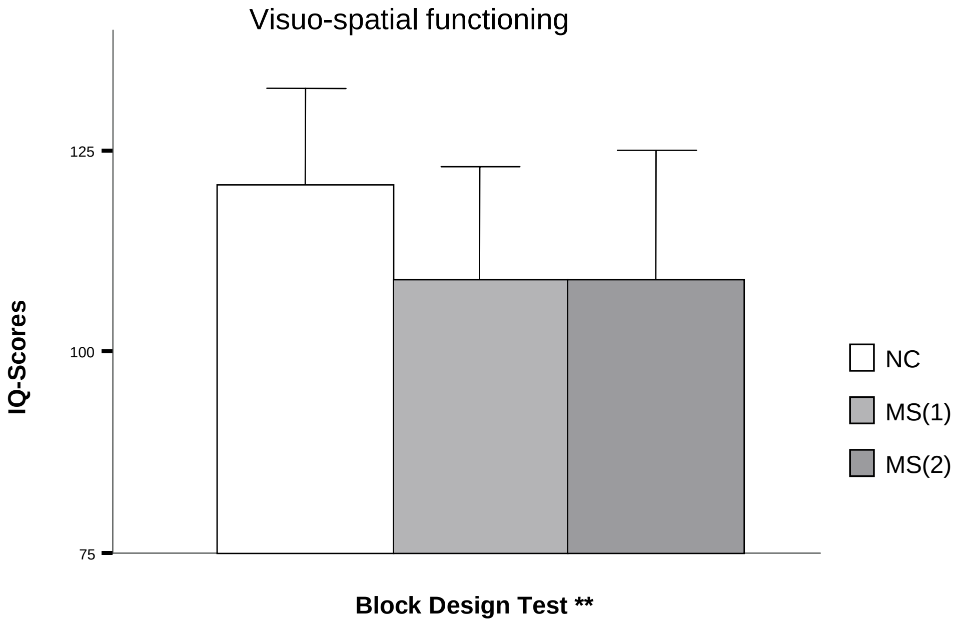

Fig. 2. Mean block design IQ-Scores (+/− SDs).

NONS: $F(2,101) = 6.14, p = 0,003$). In the paired-group comparisons, the MS(1) and MS(2) groups performed worse relative to the control group NC (MS(1): $p = 0.04$ and MS(2) $p = 0.003$, respectively, see Fig. 3).

In addition, depression (DS) was significantly correlated with other variables of depression (BL, BFS) ($r > 0.659, p < 0.001$) in both MS patient groups, but not with cognitive variables.

## 5. Discussion

In the present study, we found several differences between both groups of MS patients (EDSS 0 and EDSS 1–2) and the control group. With regard to cognitive functioning, both MS-groups performed significantly worse at visuo-constructive functions as well as short-term recognition memory of nonsense figures than did healthy women. This deficit was present in patients

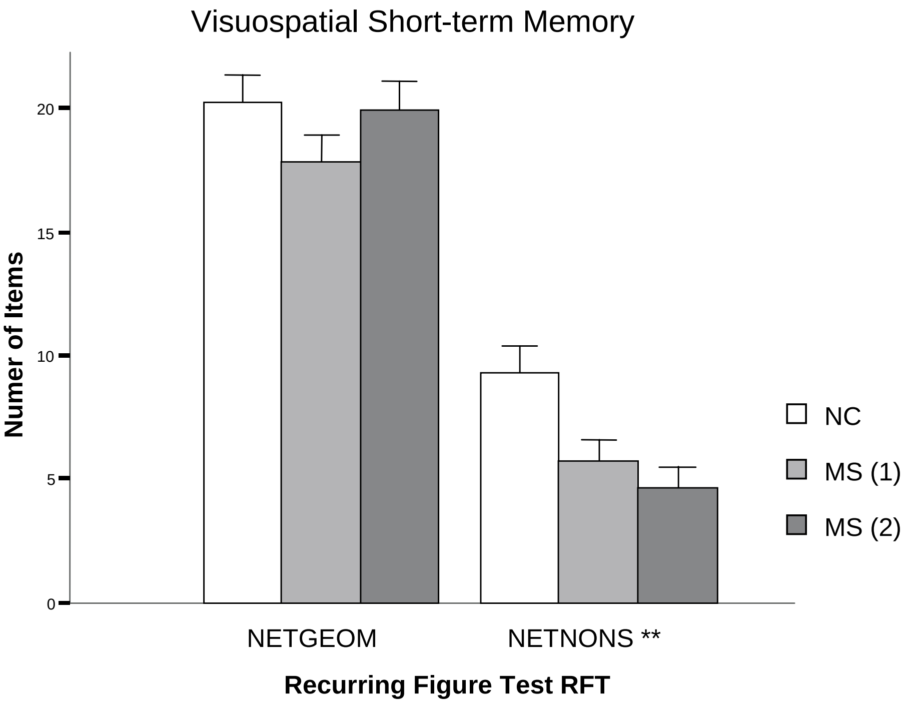

Fig. 3. Mean number (+/− SEM) of (correct minus misleadingly) remembered recurring figures of Kimura's recurring figures test (NETGEOM: geometrical figures, NETNONS: nonsense figures).

with no physical disability (EDSS 0), but it was more pronounced in patients with EDSS of 1–2, indicating a relation to physical disease progression. This is consistent with other studies on cognition, particularly those of memory, which have found impaired recall after one study trial to be a reliable and robust phenomenon when MS patients were still only mildly disabled [6,7,14,20, 21]. This disturbance, however, could be due to optic neuritis (ON), a frequent, often transient visual symptom in MS, which is reported as an initial and early symptom in a majority of patients with MS. In our patients, visual acuity was normal, although 58% had experienced at least one episode of ON. This suggests that our patients' deficit on Kimura's test cannot be related to ON, but must have a different explanation, e.g. MS lesions in the temporal lobes of the patients. This is supported by evidence that the right temporal lobe supports visual memory performance whereas the left temporal lobe supports verbal memory performance. Damage to the temporal lobes caused by MS lesions could, therefore, cause the specific kind of memory deficit we have examined in the current study [16,17].

With respect to emotional disturbances following the diagnosis of MS and the need to cope with the illness, MS seems to put a high burden of emotional distress on the usually young female patients. Depression can either be due to a reactive depression or to depressive coping [37]. However, our study was designed to exclude somatic symptoms of MS or side effects of steroid therapy (such as influences on cognition, sleep disturbances or loss of body weight) as possible causes of depression of MS patients, because only patients with EDSS 0–2 without current therapies were included. The results obtained by our study corroborated the findings regarding depression of an earlier study [8]. Although the precise pathomechanism of depression has not yet been determined, e.g. the total load of lesioned areas visible in magnetic resonance imaging do not correlate with the extent of the depression [1,31]. Berg et al. [3] have shown that in patients with higher EDSS scores a higher level of depression correlated with extent of lesioning to areas of the temporal lobe.

Table 3
Results for netgeom, netnons and net (means and SDs)

| | NC | MS(1) | MS(2) |
|---|---|---|---|
| NETGEOM | 20.25 (6.07) | 17.85 (6.21) | 19.95 (7.27) |
| NETNONS | 9.28 (6.58) | 5.70 (5.18) | 4.62 (5.42) |
| NET | 29.50 (11.76) | 23.84 (9.49) | 25.43 (9.46) |

In our study, no correlation was found between depression and recurrent figure disturbances. Our results suggested comparable deficits even in patients without disability. These findings therefore point to a rather slow development of increasing disabilities and impairments which begin very early and can only be identified when they become more severe at a later time point [4] or with even more sensitive tests.

In MS patients, depression should already be specifically assessed in the early phase of the disease, in order to integrate depression into therapeutic approaches at this stage. From a psychoneuroimmunological perspective it needs to be tested how depression and memory impairment in the early phase of MS have an impact on the further course of the disease as well as on neuroendocrine homeostasis [11]. Our study introduced easy-to-use tests, which gave supportive data for early neuropsychological impairment being part of a continuum from the very beginning of the chronic demyelinating disease process in MS. As those changes can have a substantial effect on the activities of daily living, their recognition and treatment needs early consideration [1].

## Acknowledgment

This study was supported in part by a grant from the Gemeinnützige Hertie-Stiftung, Frankfurt (GHS 274/93).

## References

[1] M.P. Amato, G. Ponziani, G. Pracucci, L. Bracco, G. Siracusa and L. Amaducci, Cognitive impairment in early-onset multiple sclerosis, Pattern, predictors, and impact on everyday life in 4-year follow-up, *Archives of Neurology* **52** (1995), 168–172.

[2] A.T. Beck, R.A. Steer and M.G. Garbin, Psychometric properties of the Beck Depression Inventory: twenty-five years of evaluation, *Clinical Psychology Review* **8** (1988), 77–100.

[3] D. Berg, T. Supprian, J. Thomae, M. Warmuth-Metz, A. Horowski, B. Zeiler, T. Magnus, P. Rieckmann and G. Becker, Lesion pattern in patients with multiple sclerosis and depression, *Multiple Sclerosis* **6** (2000), 156–162.

[4] P.A. Brex, O. Ciccarelli, J.I. O'Riordan, M. Sailer, A.J. Thompson and D.H. Miller, A Longitudinal study of abnormalities on MRI and disability from multiple sclerosis, *New England Journal of Medicine* **346** (2002), 158–164.

[5] G. Dahl, Reduzierter Wechsler-Intelligenztest (Short version of the Wechsler Intelligence test), Hain Verlag, Meisenheim, 1972.

[6] J. DeLuca, S. Barbieri-Berger and S.K. Johnson, The nature of memory impairments in multiple sclerosis: acquisition versus retrieval, *Journal of Clinical and Experimental Neuropsychology* **16** (1994), 183–189.

[7] J. DeLuca, E.A. Gaudino, B.J. Diamond, C. Christodoulou and R.A. Engel, Acquisition and storage deficits in multiple sclerosis, *Journal of Clinical and Experimental Neuropsychology* **20** (1998), 376–390.

[8] P.M. Faustmann and R.E. Ganz, Depressiveness in the relapse-free early stage of multiple sclerosis (in German), *Aktuelle Neurologie* **22** (1995), 173–175.

[9] P.M. Faustmann and R.E. Ganz, Depressiveness and memory impairment prior to significant physical disability in early multiple sclerosis, *Journal of Neurology* **244** (1997), S75.

[10] P.M. Faustmann, H.Ch. Wolf, O. Kastrup and R.E. Ganz, Bipolar affective psychosis and multiple sclerosis (in German), *Nervenheilkunde* **19** (2000), 570–574.

[11] F.W. Foley, U. Traugott, N.G. LaRocca, C.R. Smith, K.R. Perlman, L.S. Caruso and L.C. Scheinberg, A prospective study of depression and immune dysregulation in multiple sclerosis, *Archives of Neurology* **49** (1992), 238–244.

[12] R.E. Ganz and P.M. Faustmann, Functional coupling between the central-autonomic and the cognitive systems is enhanced in states after optic neuritis and early multiple sclerosis, *International Journal of Neuroscience* **86** (1996), 87–93.

[13] I. Grant, W.I. McDonald, M.R. Trimble, E. Smith and R. Reed, Deficient learning and memory in early and middle phases of multiple sclerosis, *Journal of Neurology, Neurosurgery and Psychiatry* **47** (1984), 250–255.

[14] P. Hämäläinen and J. Ruutiainen, Cognitive decline in multiple sclerosis, *The International Multiple Sclerosis Journal* **6** (1999), 51–57.

[15] W. Hartje and H. Rixecker, Kimura's recurring figures test: Standardization on a German population-sample (in German), *Nervenarzt* **49** (1978), 354–356.

[16] W.G. Honer, T. Hurwitz and D.K.B. Li, Temporal lobe involvement in multiple sclerosis patients with psychiatric disorders, *Archives of Neurology* **44** (1987), 187–190.

[17] D. Kimura, Right temporal lobe damage, *Archives of Neurology* **8** (1963), 264–271.

[18] J.F. Kurtzke, Rating neurologic impairment in multiple sclerosis, An expanded disability status scale (EDSS), *Neurology* **33** (1983), 1444–1452.

[19] L.B. Krupp, M. Sliwinski, D.M. Masur, F. Friedberg and P.K. Coyle, Cognitive functioning and depression in patients with chronic fatigue syndrome and multiple sclerosis, *Archives of Neurology* **51** (1994), 705–710.

[20] N. I. Landro, H. Sletvold and E. Gulowsen Celius, Memory functioning and emotional changes in early phase multiple sclerosis, *Archives of Clinical Neuropsychology* **15** (2000), 37–46.

[21] S.A. McIntosh-Michaelis, M.H. Roberts, S.M. Wilkinson, I.D. Diamond, D.L. McLellan, J.P. Martin and A.J. Spackman, The prevalence of cognitive impairment in a community survey of multiple sclerosis, *British Journal of Clinical Psychology* **30** (1991), 333–348.

[22] E. Millefiorini, A. Padovani, C. Pozzili, C. Loriedo, S. Bastianello, C. Buttinelli, V. Di Piero and C Fieschi, Depression in the early phase of MS: Influence of functional disability, cognitive impairment and brain abnormalities, *Acta Neurologica Scandinavica* **86** (1992), 354–358.

[23] S.L. Minden, E.J. Moes, J. Orav, E. Kaplan and P. Reich, Memory impairment in multiple sclerosis, *Journal of Clinical and Experimental Neuropsychology* **12** (1990), 566–586.

[24] S.L. Minden and R.B. Schiffer, Affective disorders in multiple sclerosis, Review and recommendations for clinical research, *Archives of Neurology* **47** (1990), 98–104.

[25] P.J. Modrego and J. Ferrández, Familial multiple sclerosis with repetitive relapses of manic psychosis in two patients (mother and daughter), *Behavioural Neurology* **12** (2000), 175–179.

[26] A. Möller, G. Wiedemann, U. Rohde, H. Backmund and A. Sonntag, Correlates of cognitive impairment and depressive mood disorder in multiple sclerosis, *Acta Psychiatrica Scandinavica* **89** (1994), 117–121.

[27] M.F. Penman, Assessing the prevalence of cognitive impairment in multiple sclerosis: Implications for patient management, *Axon* **13** (1991), 45–49.

[28] C.M. Poser, D.W. Paty, L. Scheinberg, I. McDonald, F.H. Davis and W.W. Tourtellotte, New diagnostic criteria for multiple sclerosis: Guidelines for research protocols, *Annals of Neurology* **13** (1983), 227–231.

[29] S.M. Rao, Neuropsychology of multiple sclerosis: A critical review, *Journal of Clinical and Experimental Neuropsychology* **8** (1986), 503–542.

[30] S.M. Rao, G.J. Leo and P. St Aubin-Faubert, On the nature of memory disturbance in multiple sclerosis, *Journal of Clinical and Experimental Neuropsychology* **11** (1989), 699–712.

[31] S.M. Rao, Neuropsychology of multiple sclerosis, *Current Opinion in Neurology* **8** (1995), 216–220.

[32] A.D. Sadovnik, R.A. Remick, J. Allen, E. Swartz, I.M. Yee, K. Eisen, R. Farquhar, S.A. Hashimoto, J. Hooge, L.F. Kastrukoff, W. Morrison, J. Nelson and J. Oger, Depression and multiple sclerosis, *Neurology* **46** (1996), 628–632.

[33] D. von Zerssen and D.-M. Koeller, Klinische Selbstbeurteilungs-Skalen (KSb-S) aus dem Münchener Psychiatrischen Informations-System (PSYCHIS München), Manuale, Beltz, Weinheim, 1976.

[34] D. von Zerssen, Clinical self-ratings scales (CSRS) of the Munich Psychiatric Information System, in: *Assessment of Depression*, N. Sartorius and T.A. Ban, eds, Springer Verlag, Berlin, 1986, pp. 270–303.

[35] D. von Zerssen and C. Cording, The measurement of change in endogenous affective disorders, *Archives of Psychiatry and Neurological Sciences* **226** (1978), 95–112.

[36] D. von Zerssen, F. Strian and D. Schwarz, Evaluation of depressive states, especially in longitudinal studies, in: *Psychological Measurements in Psychopharmacology*, Vol. 7, Modern Problems in Pharmacopsychiatry, P. Pichot and R. Olivier-Martin, eds, Karger, Basel, 1974, pp. 189–202.

[37] S. Warren, K.G. Warren and R. Cocherill, Emotional stress and coping in multiple sclerosis exacerbation, *Journal of Psychosomatic Research* **35** (1991), 37–47.

[38] F.A. Whitlock and M.M. Siskind, Depression as a major symptom of multiple sclerosis, *Journal of Neurology, Neurosurgery, and Psychiatry* **43** (1980), 861–865.
